# Technology-based intervention programs to promote stimulation control and communication in post-coma persons with different levels of disability

**DOI:** 10.3389/fnhum.2014.00048

**Published:** 2014-02-11

**Authors:** Giulio E. Lancioni, Andrea Bosco, Marta Olivetti Belardinelli, Nirbhay N. Singh, Mark F. O'Reilly, Jeff Sigafoos, Doretta Oliva

**Affiliations:** ^1^Department of Neuroscience and Sense Organs, University of BariBari, Italy; ^2^Department of Educational Science, Psychology, Communication, University of BariBari, Italy; ^3^Department of Psychology, “La Sapienza” UniversityRome, Italy; ^4^Department of Psychiatry and Health Behavior, Medical College of Georgia, Georgia Regents UniversityAugusta, GA, USA; ^5^Department of Special Education, University of Texas at AustinAustin, TX, USA; ^6^Department of Educational Psychology, Victoria University of WellingtonWellington, New Zealand; ^7^Lega F. D'Oro Research CenterOsimo, Italy

**Keywords:** technology-based programs, minimally conscious state, emergence from minimally conscious state, communication, stimulation, leisure

## Abstract

Post-coma persons in a minimally conscious state and with extensive motor impairment or emerging/emerged from such a state, but affected by lack of speech and motor impairment, tend to be passive and isolated. A way to help them develop functional responding to control environmental events and communication involves the use of intervention programs relying on assistive technology. This paper provides an overview of technology-based intervention programs for enabling the participants to (a) access brief periods of stimulation through one or two microswitches, (b) pursue stimulation and social contact through the combination of a microswitch and a sensor connected to a speech generating device (SGD) or through two SGD-related sensors, (c) control stimulation options through computer or radio systems and a microswitch, (d) communicate through modified messaging or telephone systems operated via microswitch, and (e) control combinations of leisure and communication options through computer systems operated via microswitch. Twenty-six studies, involving a total of 52 participants, were included in this paper. The intervention programs were carried out using single-subject methodology, and their outcomes were generally considered positive from the standpoint of the participants and their context. Practical implications of the programs are discussed.

## Introduction

Post-coma persons in a minimally conscious state and with extensive motor impairment or emerging/emerged from such a state, but affected by lack of speech and motor impairment, tend to be passive and isolated and can experience impoverished environmental input (Giacino, 1996; Elliott and Walker, 2005; Kalmar and Giacino, 2005; Wales and Waite, 2005; Whyte, 2007; Katz et al., 2009; Lancioni et al., 2010a, 2011d; Bruno et al., 2011; Bodart et al., 2013). Helping these persons improve their condition is a critical objective of any rehabilitation and care center, and any family context dealing with them (Naudé and Hughes, 2005; Hirschberg and Giacino, 2011; Wallace and Bradshaw, 2011; McNamee et al., 2012; Müller-Patz et al., 2013; Seel et al., 2013).

Studies conducted with persons in a minimally conscious state and extensive motor impairment have reported four different intervention approaches in addition to pharmacological strategies (e.g., use of amantadine; see Schnakers et al., 2008; Oliveira and Fregni, 2011; Giacino et al., 2012a,b). The four intervention approaches involve general environmental stimulation (i.e., sensory stimulation including music, and social-tactile stimulation), transcranial magnetic stimulation (i.e., a procedure that uses magnetic fields to stimulate nerve cells in the brain), deep brain stimulation (i.e., electrical stimulation of the thalamus through implanted electrodes), and technology-based programs (i.e., procedures based on the use of assistive technology to monitor responding and follow it with relevant stimulation/events) (Giacino, 1996; Magee, 2005, 2007; Pape et al., 2009; Daveson, 2010; Georgiopoulos et al., 2010; Lancioni et al., 2010a; Hirschberg and Giacino, 2011; Piccione et al., 2011; O'Neill, 2012; Scherer, 2012). Early reviews of the studies using general environmental stimulation have cast doubts as to the real effects of such an approach (Vanier et al., 2001; Lombardi et al., 2002). More recent attempts to use the same approach have suggested possible positive effects of intensive stimulation (Lotze et al., 2011) and of stimulation carried out through the use of daily objects considered relevant for the participants (Di Stefano et al., 2012).

The studies dealing with transcranial magnetic stimulation have also reached different conclusions. For example, Pape et al. (2009), Piccione et al. (2011), and Angelakis et al. (2014) reported that such stimulation produced improvements in arousal and awareness considered to be quite valuable. Manganotti et al. (2013), on the other hand, reported that only one of the six participants involved in their study had an improvement in arousal. The studies reporting on deep brain stimulation have stressed that this approach, which might prove quite relevant in terms of its overall impact, is still at the experimental stage and not free from serious risks related to the implantation and maintenance of the electrodes (Schiff et al., 2007, 2009; Giacino et al., 2012a; Yamamoto et al., 2013).

The studies reporting technology-based interventions with persons in a minimally conscious state and with extensive motor impairment varied from the others in that the technology (e.g., microswitch and electronic control device) was used as a means to enable the participants to (a) develop small/simple responses and (b) use them to manage stimulation access and social interaction requests on their own (Lancioni et al., 2010a, 2013b,c; Wallace and Bradshaw, 2011). In practice, (a) the objective of the studies was to help the participants acquire an active role, rather than simply increase their stimulation input and, possibly, their general alertness and overall activity, and (b) the results were largely positive (Lancioni et al., 2010a, 2011c, 2012b,d; McNamee et al., 2012).

The studies directed at persons emerging/emerged from a minimally conscious state, but affected by lack of speech and motor impairment varied as to whether they pursued (a) stimulation and choice, (b) communication, or (c) both communication and stimulation (Lancioni et al., 2011e,g, 2012c, 2013a). All those studies were based on the use of experimental technology solutions (e.g., microswitches and modified messaging or telephone systems) that were arranged specifically to address performance objectives and response limitations of the participants involved. The results reported by the studies were highly encouraging, opening new perspectives for rehabilitation programs directed at those participants (Lancioni et al., 2013a).

This paper provides (a) a list of the aforementioned studies using technology-based intervention programs with post-coma persons in a minimally conscious state or emerging/emerged from such a state and affected by extensive motor impairment (with specification of number and ages of participants involved and of responses and technology used; see Table 1), (b) detailed summaries of some of the studies, as illustrative examples of the programs' characteristics and results, and (c) considerations on the programs' implications. The studies (all conducted by these authors who were, to our knowledge, the only research team to assess such programs) were divided into five groups based on whether they were aimed at enabling the participants to (a) access brief periods of stimulation through one or two microswitches, (b) pursue stimulation and social contact through the combination of a microswitch and a sensor connected to a speech generating device (SGD) or through two SGD-related sensors, (c) control stimulation options through computer or radio systems and a microswitch, (d) communicate through modified messaging or telephone systems operated via microswitch, and (e) control combinations of leisure and communication options through computer systems operated via microswitch. Twenty-six studies, involving a total of 52 participants, were included in the paper. The studies were carried out using single-subject research designs (Kennedy, 2005; Barlow et al., 2009), and their results were generally considered positive from the standpoint of the participants and their context (Lancioni et al., 2010a, 2011a,d,g, 2012c,d, 2013a,b,c).

**Table 1:** **List of studies using technology-aided intervention programs to promote stimulation control and communication in post-coma patients**.

**Studies groups**	**Participants**	**Age (years)**	**Responses**	**Technology**
**BRIEF STIMULATION ACCESS THROUGH ONE OR TWO MICROSWITCHES**				
Lancioni et al., 2009a	1	26	Full eyelid closure	Optic microswitch mounted on eyeglasses' frames and linked to an electronic device that controlled brief music presentations
Lancioni et al., 2009a	1	45	Head turning and foot movement	Pressure microswitch on the chair's headrest and tilt plus pressure microswitch on the foot, both linked to an electronic device that controlled video clips and audio-recordings
Lancioni et al., 2010b	2	56 and 53	Finger and head movement for the first participant, and upward eyelid movement and hand stroking/pushing for the second participant	Touch and tilt microswitches for the first participant, and optic and touch-pressure microswitches for the second participant. The microswitches were linked to an electronic device that controlled music and video clips
Lancioni et al., 2010c	1	41	Lip movement	A two-element optic microswitch was (a) fixed at the participant's chin for monitoring lip movements and (b) linked to a device that controlled music and verbal stimuli
Lancioni et al., 2010e	2	79 and 52	Right and left head turning for the first participant, and right and left head bending for the second participant	Tilt microswitches for the first participant and optic microswitches for the second participant. The microswitches were linked to an electronic device that controlled music and prayer clips or two series of video clips
Lancioni et al., 2011d	3	67–77	Full eyelid closure for the first participant, finger movement for the second participant, and protracted eyelid closure for the third participant	Camera-based microswitch for the first participant, touch microswitch for the second participant, and optic microswitch on eyeglasses' frame for the third participant. The microswitches were linked to an electronic device that controlled brief music events. These could be combined with body massage
Lancioni et al., 2012b	1	53	Finger movement	Touch microswitch linked to an electronic device that controlled brief music events. These could be combined with body massage
Lancioni et al., 2012d	5	37–78	Protracted eyelid closure for three participants, small hand closure for the fourth participant, and toe movement for the fifth participant	Optic microswitch on the participant's cheekbone for three participants, touch-pressure microswitch for the fourth participant, and tilt microswitch for the fifth participant. The microswitches were linked to an electronic device that controlled brief music events and video clips
Lancioni et al., 2012e	3	23–60	Protracted or repeated eyelid closure	Optic microswitch on the participant's cheekbone. The microswitch was linked to an electronic device that controlled brief music events and video clips
**BRIEF STIMULATION AND SOCIAL CONTACT THROUGH ONE MICROSWITCH AND ONE SGD-RELATED SENSOR OR THROUGH TWO SGD-RELATED SENSORS**				
Lancioni et al., 2009a	1	18	Hand movement (small lifting of the hand) and toe movement	Optic sensor under the hand for one type of SGD contact calls and tilt sensor on the toe for a second type of SGD contact calls. The SGD-related sensors were linked to an electronic device that controlled the two types of calls (i.e., calls for a research assistant and calls for the mother or grandfather)
Lancioni et al., 2009b	2	35 and 60	Hand movement to reach the leg and hand movement to reach the stomach for the first participant, and small hand closure and toe movement for the second participant	Touch-pressure microswitch on the leg and pressure sensor for SGD contact calls at the stomach area for the first participant, and touch-pressure microswitch inside the hand and tilt sensor for SGD calls on the toe for the second participant. The microswitches and SGD-related sensors were linked to an electronic device that controlled brief music events or video clips and calls for the research assistant
Lancioni et al., 2009c	2	32 and 33	Repeated eyelid closure and small hand closure for the first participant, and head movement and protracted eyelid closure for the second participant	Optic microswitch held through a wire fixed on the forehead and touch-pressure sensor for SGD calls inside the hand for the first participant, and pressure microswitch in front and optic sensor for SGD calls held through a wire fixed at the temple side. The microswitches and SGD-related sensors were linked to an electronic device that controlled (a) brief music events and calls for the mother or research assistant for the first participant, and (b) video clips of sport and music and calls for the research assistant for the second participant
**STIMULATION OPTIONS THROUGH COMPUTER OR RADIO SYSTEMS AND A MICROSWITCH**				
Lancioni et al., 2010d	3	22–81	Hand movement to reach the microswitch for the first participant, repeated eyelid closure or light head movement for the second participant, and small hand closure for the third participant	A computer system with a set of 16 stimuli was used at each session. For each stimulus, the computer presented a sample of a few seconds (e.g., audio- and video-recordings of music, family members, and comedy). The participants had a pressure microswitch, an optic and a touch microswitch, and a touch-pressure microswitch, respectively, to monitor their responses. Microswitch activation within a few seconds from the end of the sample led the computer to present about 20 s of that stimulus. Any new microswitch activation soon after the end of a 20-s stimulus presentation led to 20 additional seconds of that stimulus. No microswitch activation after a sample or following a 20-s stimulus period led the computer to present the next sample. The same conditions applied until the end of the session
Lancioni et al., 2010g	1	57	Vocalization/laughter	A computer system with a set of 16 stimuli was used at each session. For each stimulus, the computer presented a sample of a few seconds (i.e., audio-recording of a comic sketch). The participant had a voice detecting microswitch to monitor his responses. Microswitch and computer system worked as described for the previous study
Lancioni et al., 2011e	3	42–62	Repeated eyelid closure for one participant, and protracted eyelid closure for the other two participants	A computer system with a set of 16 stimuli was used at each session. For each stimulus, the computer presented a sample of a few seconds (i.e., video and music). Participants had an optic microswitch to monitor their eyelid responses. Microswitch and computer system worked as described for the previous studies
Lancioni et al., 2012d	3	49–84	Small hand closure for all three participants	A computer system with a set of 19 stimuli was used at each session. Some of the stimuli concerned caregiver's maneuvers that the participants could desire/need (e.g., having the face washed and the tracheal cannula cleared). Each participant had a pressure microswitch inside the hand. Microswitch and computer system worked as in the previous studies
Lancioni et al., 2013b	1	59	Smile	A computer system with a set of 16 stimuli was used at each session. For each stimulus, the computer presented a sample of a few seconds (i.e., audio-recording of a comic sketch). The participant had a webcam microswitch to monitor his smile responses. Microswitch and computer system worked as in the previous studies
Lancioni et al., 2011d	1	51	Hand movement to reach the microswitch	A modified radio device was used with a computer, an amplified MP3, and a pressure microswitch. Ten stations were preselected. At the start of each session, the radio was tuned on one of those stations. Any microswitch activation set it on the next station. Microswitch activation when it was set on the tenth station turned it off. A new microswitch activation after that turned it on again. After periods of 3 min spend on the same station, the MP3 asked whether he was enjoying that station
Lancioni et al., 2012c	3	44–71	Small hand closure for the first participant, toe movement for the second participant, and slight head movement for the third participant	The radio device, computer, and MP3 were as in the previous study. The participants had a touch-pressure microswitch, a tilt microswitch, and an optic microswitch, respectively. All conditions were as in the previous study
**COMMUNICATION THROUGH MODIFIED MESSAGING AND TELEPHONE SYSTEMS AND A MICROSWITCH**				
Lancioni et al., 2010f	2	39 and 50	Hand movement to reach the microswitch	Optic microswitch at the right ear for one participant, and pressure microswitch on the chair's tray for the other participant. First microswitch activation triggered the messaging system to list the persons available. Second microswitch activation served to select one of the persons. Third microswitch activation served to to select a message out of those listed by the system and send it to the person. Microswitch activation served also to have incoming messages read out
Lancioni et al., 2011g	2	43 and 54	Prolonged eyelid closure or hand movement to activate the microswitch	Optic microswitch on the participant's cheekbone. For one participant, this was later replaced by pressure microswitch. First microswitch activation triggered the messaging system to list the groups of people available for messages. Second microswitch activation served to select a group. Third and fourth activation served to select a person and the message to send, respectively. Microswitch activation served also to have incoming messages read out
Lancioni et al., 2011a	2	40 and 44	Hand movement to reach the leg for the first participant, and small hand closure for the second participant	Optic microswitch on the leg for one participant, and touch-pressure microswitch inside the hand for the other participant. Conditions were similar to those of the previous study
Lancioni et al., 2013d	2	23 and 36	Mouth opening for one participant, and hand movement to reach the microswitch for the other participant	Camera-based microswitch for one participant, and wobble microswitch for the other participant. Microswitch activations served to (a) have the persons available for call and their voices presented by the computer-aided telephone system, (b) select one of the persons, and (c) have the system place a call to him or her
**LEISURE AND COMMUNICATION OPTIONS THROUGH COMPUTER SYSTEMS AND A MICROSWITCH**				
Lancioni et al., 2011f	1	40	Small hand closure	A computer system was available with four cells being in turn scanned on its screen (i.e., interacting with parents and research assistant, watching a film segment, listening to a song, and watching a television program segment). The participant could select any cell by activating the touch-pressure microswitch inside her hand while the cell was being scanned. Cell selection opened a new screen with various, related options. These options were automatically scanned. Selection of one option led the computer to activate such option (i.e., call the person selected for interaction, play a song, a film or a television segment). Then the system automatically reset on the first screen with the four cells
Lancioni et al., 2013a	2	44 and 24	Hand movement to reach the microswitch	A computer system was available that allowed the participants to choose (a) initially among music, videos and caregiver's maneuvers, and (b) subsequently among those three options plus messaging. The options were shown through screen cells that the participants could select by activating a touch-pressure microswitch. Selection of one of the first three options opened a new screen with multiple alternatives. Selection of one of them worked as in the previous study. Selection of the messaging option started a sequence such as that described in the studies focused on messaging (see above)
Lancioni et al., 2013c	2	55 and 56	Small hand closure for the first participant, and small head movement for the second participant	A computer system was available that allowed the participants to choose among music, videos, caregiver's maneuvers, messaging, and placing phone calls. These options were presented on the computer screen (i.e., cells being automatically scanned). The participants could select an option by activating the microswitch while the related cell was being scanned. A touch-pressure microswitch inside the hand was used for the first participant; an optic microswitch under the chin was used for the second participant. Selection of one of the first three options worked as in the previous two studies. Selection of the messaging or phoning option started a sequence such as those described in the studies focused on those communication forms (see above)

## Brief stimulation access through one or two microswitches

Intervention programs with one microswitch are generally used for post-coma persons in a minimally conscious state (also in a low/*minus* level of the state; see Bruno et al., 2011) and extensive motor impairment. Those programs are characterized by the use of (a) a sensor (microswitch) that can detect a small and feasible response of the participant and (b) an electronic device, which is connected to the sensor and regulates the presentation of brief periods of stimulation contingent on the participant's responding. Indeed, the microswitch is a tool that allows the participant to produce an environmental change (i.e., occurrence of relevant stimulation) with a minimal response (e.g., eyelid closure or finger movement). In other words, the microswitch allows the participant to develop a previously irrelevant (non-functional) response and increase its frequency to improve his or her level of stimulation and, possibly, his or her wellbeing (Lancioni et al., 2010a).

For example, Lancioni et al. (2010c) implemented an intervention program with a 41-year old woman who had incurred brain injury and coma subsequent to a car accident 15 years prior to the study. She had been considered to be in a vegetative state for many years and eventually judged to be at the lower end of the minimally conscious state. She was lying in bed with head, trunk, and limbs static. Her mouth was semi-open and, intermittently, lip movements were observed. These movements consisted of the lips moving further apart or closer together. The microswitch developed for this response involved two optic sensors attached to a metal support, which was fixed to the participant's chin. The sensors were directed at the upper lip and mouth cavity, respectively. A further separation of the lips caused the upper sensor to point to the mouth cavity rather than to the upper lip and this led to microswitch activation. Similarly, moving the lips closer caused the lower sensor to point to the lips rather than to the mouth cavity and this also led to microswitch activation. Every microswitch activation triggered an electronic control device that allowed the participant to access music or familiar voices for 10–15 s during the intervention phases of the study. Intervention sessions as well as baseline sessions (i.e., sessions in which lip movements and microswitch activation did not provide stimulation) lasted 10 min. The results showed that the response frequency was about seven (but with a declining trend) during the first baseline phase and about 15 during the first intervention phase. The second baseline and second intervention phase largely replicated the results of the first two phases.

Intervention programs with two microswitches (each used for one specific response) can be implemented with post-coma persons in a higher range of the minimally conscious state (i.e., persons who can be more prone to response engagement; Lancioni et al., 2010a). An initial baseline is carried out on both responses/microswitches selected for use. Then, the intervention concentrates on the first response/microswitch. Once the participant's responding has increased, a new baseline and an intervention phase are carried out on the second response. Subsequently, the two microswitches may be available (a) alternatively so that the participant can maintain both responses by rotating them over different sessions, in which different types of stimulation are available to them, or (b) simultaneously within the sessions so that the participant can choose between responses and types of stimulation in a totally independent manner.

Lancioni et al. (2010b) carried out an intervention program along the aforementioned lines with two participants of 56 and 53 years of age. The responses selected for the first participant consisted of finger and head movement. The microswitches for those responses were a touch-sensitive pad and a mini tilt device, respectively. The responses selected for the second participant consisted of upward eyelid movement and hand stroking/pushing. The microswitches for those responses were an optic sensor fixed on the participant's forehead and pointing to his eyelid and a touch-pressure pad. Intervention on the responses was carried out sequentially (i.e., as described above) through sessions lasting 5 min. Once the intervention on the second response was completed, sessions targeting one response were alternated with sessions targeting the other response. The mean response frequencies during baseline were about or below five per session for the first participant and below six per session for the second participant. Their mean frequencies during intervention varied between 11 and 14.

## Brief stimulation and social contact through one microswitch and one SGD-related sensor or through two SGD-related sensors

The importance of microswitch-aided programs for persons with pervasive disabilities can hardly be overstated. They are indeed critical to promote the acquisition and consolidation of adaptive/functional responses that extend the person's repertoire and empower him or her to access relevant environmental events independently. One thing those programs cannot help the person to do is to call for caregiver attention. This can become increasingly important for the person as his or her alertness and engagement improve. To deal with this requirement, programs can be developed that include a microswitch and an SGD-related sensor or even two SGD-related sensors (see Table 1). The SGD-related sensors are comparable to the sensors used as microswitches. Their activation, contrary to that of the microswitches, does not cause access to specific stimulus events but produces a verbal output from the SGD, that is, a call to the caregiver for some form of attention and contact. In relation to the call, the caregiver may intervene with verbal attention or with verbal attention, physical contact, and the sharing of stimulus material.

Lancioni et al. (2009b) implemented an intervention program that included the combination of a microswitch and an SGD-related sensor with a man and a woman of 35 and 60 years of age, respectively, who had been diagnosed in the high range of the minimally conscious state. Both participants also presented extensive motor impairment that precluded them any control of environmental events and lack of speech. The program involved several daily sessions, which lasted 7 and 5 min for the two participants, respectively. The responses selected for the man consisted of touching the left leg (for activating the microswitch, which was a touch-pressure device) and touching the stomach (for activating the SGD-related sensor, which was a pressure device). The responses selected for the woman consisted of a small hand closure (for activating the microswitch, which was a touch-pressure device fixed in the palm of her right hand) and a movement of the big toe of her right foot (for activating the SGD-related sensor, which consisted of mini tilt devices). Activating the microswitch allowed the participants to have direct access to brief periods of preferred music or video-clips. Activating the SGD-related sensor caused the emission of a verbal output (i.e., a call to the caregiver). In relation to the call, the caregiver approached the participant, talked to him or her, and showed photographs and videos. The implementation of the program involved three phases. The first phase was aimed at teaching the participants to use the microswitches, that is, to perform the response that was required to access relevant environmental stimuli. The second phase was aimed at teaching the participants to use the SGD-related sensors, that is, to perform a different type of response, which was required to call the caregiver and obtain her attention. The third phase was aimed at providing the participants with choice opportunities. In practice, the microswitches and the SGD-related sensors were simultaneously available to the participants within each session and they could choose freely between the two. Both participants progressed successfully through all phases of the program. During the last phase, their mean cumulative response frequencies per session were 12 and 13, indicating high levels of engagement and preferences.

## Stimulation options through computer or radio systems and a microswitch

Participants who are in the process of emerging from a minimally conscious state (or have already emerged from such a state) would probably enjoy the opportunity to choose among various environmental stimuli. However, this opportunity could be quite elusive when the participants are affected by extensive motor impairment and lack of speech, unless appropriate technology-aided programs are implemented. Two such programs have been developed (Lancioni et al., 2011d, 2012c,d). One of them is relying on the use of (a) a computer system that presents samples of environmental stimuli (which can also involve visual-auditory clips of caregiver's maneuvers; see below), (b) a microswitch with which the participants can express their stimulus/maneuver selection or lack thereof, and (c) a software ensuring that a selection response is followed by a preset play period of that stimulus (or application of the chosen maneuver) and a non-selection response is followed by the presentation of the next stimulus sample available in the sequence. The software also ensures that a selection (microswitch) response carried out shortly after the end of the stimulus' play period produces a new play period of that stimulus (i.e., the same or the following segment or the stimulus). For example, Lancioni et al. (2012d) carried out such a program with three participants of 49–84 years of age, who were diagnosed as emerging from a minimally conscious state. A set of 19 stimuli was programmed for each session. Twelve of them were considered to be preferred events for the participants (e.g., songs and videos), four concerned caregiver's maneuvers that the participants could desire/need (e.g., face washing and tracheal cannula clearing), and three were considered non-preferred events (e.g., distorted sounds). Purposeful choice behavior would be implied if the participants showed a (near) zero selection of these last stimuli. The participants had a pressure microswitch fixed in the palm of their hand and could activate it with a small hand-closure response. During the baseline phase, the participants' mean frequencies of responses per session were below seven concerning the preferred stimuli and about or below two concerning the caregiver's maneuvers. During the intervention phase, their mean frequencies of responses per session were 55, 36, and 24, respectively, concerning the preferred stimuli and between three and five concerning the caregiver's maneuvers. All participants asked for repetitions of some of the stimuli chosen. Their responses in relation to the non-preferred stimuli were only sporadic and mainly occurred during the baseline and the start of the intervention phase.

The second type of technology-aided program to allow access to (choice among) various stimulation options relies on the use of (a) a digital radio system in which 10 stations are preselected, (b) a microswitch through which the participants can change stations and turn “off” and “on” the system, (c) an amplified MP3 player with recorded verbal messages, and (d) software control. Each microswitch activation causes the system to tune onto the following station of the sequence. If the system is tuned on the tenth station, microswitch activation causes it to turn off. If the system is off, microswitch activation turns it on and tunes it on the first station of the sequence. After a 3-min period spent on a same station, the MP3 is emitting a sentence asking the participant whether he or she is enjoying that station (i.e., a check considered useful to limit drops in attention/alertness). Lancioni et al. (2012c) carried out such a program with three participants of 44–71 years of age, who had emerged from a minimally conscious state but presented with extended physical impairment and lack of or minimal speech. The responses they used to operate the radio were a small hand closure, a toe movement, and a small head movement, respectively. The microswitches available for those responses were a touch-pressure sensor inside the hand, a tilt device attached to the toe, and an optic sensor under the chin, respectively. During the intervention phase, all participants (a) showed a variety of station changes per session, (b) ended the sessions independently (by turning off the system) after mean periods of close to 30 min, and (c) displayed preferences for certain stations closely identified with specific matters (e.g., sport and news or religion).

## Communication/contact through modified messaging and telephone systems and a microswitch

The possibility of communicating with family members and friends when they are not directly present can be particularly relevant for persons who have recovered consciousness but are affected by extensive motor impairment and lack of speech (i.e., persons who would have strong interests in keeping informed about the daily life of their family and friends and in re-establishing a solid contact with them, but have no means to do so). A form of contact with family and friends when not directly present could also be relevant for persons in the process of emerging from the minimally conscious state. For these latter persons, the contact may have less of an informative/cognitive meaning and more of an emotional/affective value. In the attempt to help the first group of persons, technology-aided messaging programs have been developed, which allowed the persons to choose the partner with whom to communicate, to select the message to send him or her from among a variety of messages available, and to have any incoming message read out to them (Lancioni et al., 2011g). It was realized that the use of preprogrammed messages would be a compromise that could only partially satisfy the communication and emotional needs of the participants. At the same time, it was also envisaged that such messages would prompt the partners to respond in an elaborate and personal way and thus enhance communication and relation/closeness between them and the participants.

Lancioni et al. (2011a) implemented a technology-aided messaging program with two participants of 40 and 44 years of age who had adequate receptive language skills, but did not possess any speech or could laboriously (and slowly) utter only a few words. The technology involved a computer, a communication modem, a microswitch, and specific software. The program involved 10-min sessions during the baseline phases and 20- or 30-min sessions during the intervention phases. The microswitches consisted of an optic device on the participant's leg (which was activated by a hand movement covering it) or a touch-pressure device inside the participant's hand (which was activated by a small hand closure). The initial microswitch activation triggered the computer to verbally present the groups of persons (e.g., friends) to whom messages could be sent. The participants' selection of one of the groups (i.e., by microswitch activation) led the computer to present the names of the persons included in that group. The participants' selection of one of the persons (i.e., by microswitch activation) led the computer to present the messages available (i.e., topics and related messages). Selection of a message led the computer to send it to the person previously selected. An incoming message was signaled to the participants by beeps and verbal announcements. Microswitch activation led the computer to read out the message and the name of the sender. Data showed that during the baseline phases (during which the technology was not available), the participants did not send out or listen to incoming messages independently. During the intervention phases, the messages sent out and listened to independently were above three and two, respectively, for the participant with 20-min session and above 15 and 5, respectively, for the participant with 30-min sessions.

In an effort to help two participants emerging from a minimally conscious state and affected by extensive motor disability and lack of speech, Lancioni et al. (2013d) developed a program aimed at allowing them to make telephone contacts with family members and friends. The participants were 23 and 36 years old. The technology involved a computer system with sound amplifier, a communication modem, a microswitch, and specific software. The microswitches consisted of a camera-based device (which was activated by mouth opening) and a wobble device (which was activated by small hand contact). At the start of the sessions, the computer system asked the participants whether they wanted to call somebody and then presented the names and voices of the persons available for call, one at a time. If the participant activated the microswitch shortly after hearing a name/voice, the system placed a call to that person. The person receiving a call would talk to the participant affectionately and happily. After the end of the first call, the system would repeat the same procedure until all names available for a call had been presented or a specific time had elapsed. The results indicated that during baseline phases (i.e., when the participants did not have the aforementioned microswitches) no independent phone calls were placed. During intervention phases, both participants (a) made several calls in sessions of about 15 and 10 min, (b) showed increases in indices of happiness, and (c) showed preferences about the persons called.

## Leisure and communication options through computer systems and a microswitch

Persons who have recovered their consciousness but lack motor abilities and speech ultimately need to have communication/contact opportunities (as mentioned above) as well as recreational opportunities (i.e., the possibility to engage in leisure activities that they enjoy). Indeed, the combination of the two types of opportunities could help them spend their time in a more active and self-fulfilling manner, with possibly positive implications for their overall quality of life and the satisfaction of their family, staff, and friends (Ripat and Woodgate, 2011). Obviously, these opportunities cannot be offered without the use of support technology adapted to their characteristics (Bauer and Elsaesser, 2012; Lancioni et al., 2013b). Efforts in this direction have recently led to the development of technology-based programs aimed at promoting/supporting leisure engagement and communication (see Table 1). Lancioni et al. (2013c) implemented one such program with two participants of 55 and 56 years of age who were affected by extensive motor disabilities and lack of speech except for “yes” or “yes and no” sounds. The technology involved a computer system with screen and sound amplifier, a communication modem, a microswitch, a headset with microphone, and specific software. The microswitches consisted of a pressure device inside the hand (activated through small hand closure) for the first participant and an optic sensor under the chin (activated by a small head movement) for the second participant. The computer screen showed pictorial images of “songs,” “videos,” “caregiver's maneuvers,” “messaging,” and phone calls.” Those images were scanned in succession and the participants could choose any of them by activating the microswitch while it was scanned. If they chose the songs, videos, or caregiver's maneuvers, a new set of eight pictorial images related to the option chosen appeared on the screen. Each of the images was automatically scanned or scanned and verbally named. If the participants selected a song or video, the computer system played it for about 3 min (but the participants could interrupt it at any time by microswitch activation). If the participants chose a caregiver's maneuver, the computer system verbalized it so that the caregiver could respond to it. If the participants chose the messaging option, the computer system presented a sequence such as that described above to enable them to select the person to whom they wanted to send the message as well as the type of message the wanted to send out. If the participants chose the phone calls option, the computer system enacted a sequence similar to that described in the previous section of this paper so that the participants could successfully place a phone call to the person that they wanted to contact. During the baseline phases (with 10-min sessions in which the participants did not have the aforementioned microswitches), no independent performance was recorded. During the intervention phases (involving 20-min sessions), the mean frequencies of options selected per session were above six and 10 for the two participants, respectively. Choices concerning songs, videos, and caregiver's maneuvers accounted for about half of the total. The remaining choices concerned messaging and phone calls, with one participant showing a higher frequency of phone calls and the other a higher frequency of messaging events.

## Considerations on the programs and their implications

The first consideration one can make is that technology-based intervention programs may be of critical importance for helping persons in a minimally conscious state or emerging/emerged from such state but affected by extensive motor and communication disabilities (Casey, 2011; De Joode et al., 2012; Scherer, 2012). Indeed, these persons would have no real chances to display an active role and self-determination, and to pursue relevant stimulation and communication opportunities if the programs were not available. The programs presented above varied substantially based on the level of the persons for whom they were used and on the objectives targeted for those persons.

The programs developed for persons in a minimally conscious state were predominantly based on a single microswitch/response and directed at promoting positive engagement and stimulation control, with consequent improvement of the persons' alertness/arousal levels. In some cases, the objective was extended to (a) choice between responses and stimulation events (i.e., with the use of two microswitches) and (b) choice between specific sensory stimulation and social contact (i.e., with the use of a microswitch and an SGD-related sensor). One could argue that any of these goals can be seen as particularly relevant within rehabilitation contexts in charge of these persons (Spivey, 2007; Eifert et al., 2013; Giovannetti et al., 2013). Programs with a single microswitch/response might also be viewed as functional diagnostic (i.e., learning assessment) tools for persons with a label of unresponsive wakefulness syndrome (e.g., Bosco et al., 2010; Lancioni et al., 2011b). In this capacity, they could be practical supplements to other diagnostic strategies, such as neuro-imaging techniques and evaluations of event related potentials (Bagnato et al., 2013; Bodart et al., 2013; Fingelkurts et al., 2013; King et al., 2013; Owen, 2013).

The possible benefits of the programs may be more easily appreciated in view of their affordable costs. Indeed, most of them may be arranged through the use of a simple microswitch (whose cost might vary between less than 100 and about 200 US dollars), an interface, and a portable computer with minimal software.

The programs developed for persons emerging/emerged from a minimally conscious state have been aimed at promoting (a) access to multiple stimuli and possibly caregiver's maneuvers through structured stimulus presentations and choice procedures, (b) facilitated radio usage, (c) forms of communication and contact via messaging and phone calls, and (d) combinations of leisure and communication activities. Programs dealing with multiple stimuli and caregiver's maneuvers can be considered particularly relevant to enrich the persons' input opportunities and increase their alertness and attention to (interaction/communication with) the environment (Fischer et al., 2008). The participants are not merely required to produce a response to access positive environmental stimulation or desired/needed maneuvers. Rather, they are to produce their response in relation to such stimuli/maneuvers (i.e., after the presentation of their samples or visual-verbal clips or immediately after their occurrences). Program sessions could be flexible in duration based on the persons' interest levels or general conditions. In fact, the persons could ask (a) for most stimuli and maneuvers as well as many repetitions of several of the stimuli on some days and in relation to specific sets of stimuli and maneuvers and (b) for fewer stimuli and repetitions on other days or with other stimuli/maneuvers. The programs could be set up with relatively modest costs (about 2000 US dollars), thus their use could be accessible for most rehabilitation and home contexts (Hubbard Winkler et al., 2010; Wallace, 2011).

Programs supporting the use of a radio are quite straightforward and may represent a viable and functional option that adds to the daily occupational (leisure) activities available to the participants. Such an option could be considered important and practical because (a) it would provide the participant exposure to a variety of stimulation, information, and communication inputs in a relatively simple and highly controllable manner, (b) it would represent a leisure engagement with highly normalizing value (as it constitutes a form of engagement largely practiced also by persons without disabilities), and thus likely to improve the social image of the participant within his or her context, and (c) it could be arranged with relatively modest costs (i.e., about or less than 1000 US dollars).

Programs allowing the use of messaging or phone calls may be viewed as highly functional for enhancing communication and social connections between the participants and people/partners relevant to them. This could be considered a very significant aspect especially for those participants who have very limited access to their relevant partners because of those partners' distance (i.e., due to work commitments or living arrangements). Contacting those partners could represent for the participants an enriching opportunity and a way to re-establish forms of emotional connection. The partners could improve their perception of the participants and thus upgrade the level of involvement with them with increases in attention and overall engagement time and quality (Casey, 2011; Scherer, 2012; Leonardi et al., 2013). Setting up the technology for a messaging program or a phone calls program would have an estimated cost of about 2000 US dollars.

Programs combining leisure and communication opportunities seem to be most suitable for persons with relatively high levels of functioning, in spite of their motor disabilities and lack of or minimal speech. These programs are quite comprehensive and include/expand the features of the previous programs. Indeed, the persons can access (choose among) a variety of leisure materials, can call for caregiver's maneuvers, and can use messaging and phone calls. The availability of a technology that ensures all the aforementioned options can provide the participants a fulfilling experience in terms of leisure engagement, and communication/contact with relevant partners. One would expect care and rehabilitation centers to find the costs needed for setting up such a program (approximately 3000 US dollars) agreeable in light of its potential benefits for the participants (Hubbard Winkler et al., 2010; Scherer, 2012).

## Conclusion

Technology-based intervention programs may be of critical importance for helping persons in a minimally conscious state or emerging/emerged from such state but affected by extensive motor and communication disabilities. Those programs may differ quite widely and help persons with different levels of disabilities achieve what is most immediately relevant for them. Extending the use of the programs to additional individuals, also through the involvement of new research groups, would be essential to (a) determine their general applicability and dependability and (b) identify ways of upgrading them (Kennedy, 2005; Barlow et al., 2009; Goodwin, 2010). The upgrading process should lead one to build new technology solutions that could be easily adjusted to the characteristics of the participants and thus have wider applicability and effectiveness (Bauer and Elsaesser, 2012; Lancioni et al., 2012a; Posatskiy and Chau, 2012; Shih et al., 2012). New research could also be aimed at determining participants' satisfaction as well as caregivers and rehabilitation staff's level of approval (Callahan et al., 2008; Jumisko et al., 2009; Scherer et al., 2011). These two types of assessment would help (a) identify the perception/views that direct users and their closest associates have of the programs and (b) include such views in the development of new intervention solutions (Lancioni et al., 2006; Callahan et al., 2008; Barlow et al., 2009; Ripat and Woodgate, 2011).

### Conflict of interest statement

The Associate Editor, Dr. Thomas Huenefeldt declares that, despite having collaborated with the author Dr. Marta Olivetti Belardinelli, the review process was handled objectively and no conflict of interest exists. The other authors declare that the research was conducted in the absence of any commercial or financial relationships that could be construed as a potential conflict of interest.
